# Predictors for de novo stress urinary incontinence following pelvic reconstruction surgery with transvaginal single-incisional mesh

**DOI:** 10.1038/s41598-019-55512-0

**Published:** 2019-12-16

**Authors:** Pei-Chi Wu, Chin-Hu Wu, Kun-Ling Lin, Yiyin Liu, Zixi Loo, Yung-Chin Lee, Cheng-Yu Long

**Affiliations:** 1 Department of Obstetrics and Gynecology, National Taiwan University Hospital, Taipei, Taiwan; 2 Department of Obstetrics and Gynecology, Kaohsiung Medical University Hospital, Kaohsiung Medical University, Kaohsiung, Taiwan; 3 Department of Obstetrics and Gynecology, Kaohsiung Municipal Siaogang Hospital, Kaohsiung Medical University, Kaohsiung, Taiwan; 4 Department of Urology, Kaohsiung Municipal Siaogang Hospital, Kaohsiung Medical University, Kaohsiung, Taiwan; 5 Department of Urology, Faculty of Medicine, College of Medicine, Kaohsiung Medical University, Kaohsiung, Taiwan; 6 Graduate Institute of Medicine, College of Medicine, Kaohsiung Medical University, Kaohsiung, Taiwan

**Keywords:** Outcomes research, Urethra

## Abstract

The study aims to identify predictors for de novo stress urinary incontinence (SUI) following Elevate mesh surgery. A total of 164 women who underwent Elevate mesh surgeries between November 2011 and February 2014 in a single center were included. Seventy-three women were excluded due to preoperative incontinence or concomitant mid-urethral sling surgery. Fourteen others were excluded due to incomplete medical records. Fisher’s exact test and χ^2^ test were applied. The univariate logistic regression was used for odds ratios. Of the 77 continent women, 24 (31.2%) experienced de novo SUI after the operation. Significantly more women with de novo SUI were over the age of 64 years (75.0% vs. 47.2%, *p* = 0.023, OR 3.36, 95% CI 1.15–9.79). Preoperative occult urodynamic stress incontinence (29.2% vs. 3.8%, *p* = 0.003, OR 10.0, 95% CI 2.0–50.0) and previous SUI history (41.7% vs. 7.6%, *p* = 0.001, OR 9.1, 95% CI 2.38–33.3) were 2 other predictors of de novo SUI postoperatively. In conclusion, age over 64 years old, occult urodynamic stress incontinence, and previous history of SUI are 3 significant predictors for de novo SUI following the single-incision mesh surgeries.

## Introduction

The lifetime risk of surgery for pelvic organ prolapse (POP) in the general female population was 19%^1^. About 40–50% of women with POP report urinary incontinence before surgeries^2^. In previously continent women, de novo stress urinary incontinence (SUI) may occur in approximately a quarter of those who receive pelvic reconstruction surgeries^2–4^. The incontinence symptom may be so bothersome that those women would need secondary surgeries. Studies revealed evidence that prolapse surgery combined with anti-incontinence surgery reduces the risk of postoperative SUI^5,6^; whereas, others warn the risk of difficulty voiding, urinary urgency or urge incontinence, urinary tract injuries and more medical costs in combined surgery^3,7–9^.

If we can selectively perform concomitant anti-incontinence surgery only for women high risk of de novo SUI, we can provide a treatment strategy with high efficacy, high satisfaction, and reasonable cost. Although the mechanism of de novo SUI is not fully understood, urethral kinking or external urethral compression may be the underlying cause^10^. Different surgical methods have different predictors for de novo SUI^11,12^, resulting in different de novo SUI rate^13^.

Elevate mesh is the next generation of the Perigee system, using type I polypropylene Intepro Lite mesh and single vaginal incision procedure that reaches obturator fascia and sacrospinous ligament. Lo, and his colleagues conducted a study mainly focusing on the predictors of de novo SUI after POP surgeries, including trans-obturator 4-arm mesh and native tissue^12^. However, there is limited data to evaluate the preoperative parameters in predicting postoperative SUI after single-incision mesh (SIM) surgery. In this study, we attempt to identify the predictive factors for de novo SUI following Elevate mesh placement.

## Results

Of the 77 continent women, 24 (31.2%) experienced de novo SUI after the operation. Demographic data were obtained in the 77 women including age, parity, body mass index (BMI), menopausal status, number of them under hormone replacement therapy (HRT), smokers, underlying diseases such as hypertension and diabetes mellitus, and previously received hysterectomy, pelvic reconstruction surgeries and anti-incontinence surgeries (Table 1). Surgical procedures performed are listed in Table 2. Of the 77 women, 24 (31.2%) experienced de novo SUI after the operation, and 53 women (68.8%) were continent postoperatively during follow-up. The Elevate mesh surgeries provided successfully anatomical correction of the POP of all women, as shown in Table 3.

**Table 1:** Demographic characteristics of continent women with pelvic organ prolapse (n = 77).

Age (years)	65.3 ± 9.9
Parity	3.1 ± 1.2
BMI (kg/m^2^)	24.0 ± 3.4
Menopause	73 (94.8)
Current hormone therapy	3 (3.9)
Current smoker	1 (1.3)
Diabetes mellitus	11 (14.3)
Hypertension	31 (40.0)
History of hysterectomy	14 (18.2)
History of POP and/or SUI surgery	7 (9.1)

^†^Data are given as mean ± standard deviation or n (%).

^‡^BMI: body mass index; POP: pelvic organ prolapse; SUI: stress urinary incontinence.

**Table 2:** Surgical procedures performed in this study (n = 77).

Procedures	n (%)
Anterior repair with mesh	44 (57.1)
Anterior and posterior repair with mesh	25 (32.5)
Posterior repair with mesh	8 (10.4)
Concomitant vaginal hysterectomy	48 (62.3)

**Table 3:** Pelvic organ prolapse quantification (POP-Q) values before and after surgery (n = 77).

POP-Q parameters	Pre-operative (n = 77)	Post-operative (n = 77)	*p* value^*^
Aa	2 (−2 ~ 3)	−2 (−3 ~ −1.5)	<0.001
Ba	3 (−2 ~ 8)	−2 (−3 ~ −1.5)	<0.001
C	1.5 (−7 ~ 8)	−7 (−9 ~ −3)	<0.001
Ap	0 (−1 ~ 3)	−2 (−3 ~ −1)	<0.001
Bp	1 (−2 ~ 8)	−2 (−3 ~ −1)	<0.001
TvL	9 (−7 ~ 12)	−8 (−6 ~ 10)	<0.051

^*^The Wilcoxon signed-rank test was performed for comparison between pre-operative and post-operative POP-Q parameters.

^†^Data are given as median (range) or n (%).

### Comparison between the postoperative continent and incontinent women

Compared to postoperative continent women, de novo SUI were reported more commonly in women over the age of 64 years (75.0% vs. 47.2%, *p* = 0.023) (Table 4). Besides, women with preoperative occult urodynamic stress incontinence (29.2% vs. 3.8%, *p* = 0.003, OR 10.0, 95% CI 2.0–50.0) and previous history of SUI (41.7% vs. 7.6%, *p* = 0.001, OR 9.1, 95% CI 2.38–33.3) reported higher rate of de novo SUI postoperatively (Table 5). There was no statistical difference between the two groups in parity, BMI, HRT use, smoking status, underlying disease, surgical history, preoperative POP stage, or operative procedures. Interestingly, there was also no statistical difference between the two groups in preoperative urodynamic parameters evaluation for BOO (37.5% vs. 43.4%, *p* = 0.7) and maximum urethral closure pressure (MUCP) drop after POP reduction with vaginal gauze roll (33.3% vs. 30.2%, *p* = 0.73).

**Table 4:** Analysis of clinical features in de novo stress urinary incontinence (SUI) group (n = 24) and no SUI group (n = 53).

	De novo SUI group (n = 24)	No SUI group (n = 53)	*p* value^†^	OR (95% CI)^‡^
**Age (years)**				
<64	6 (25.0)	28 (52.8)		1 (reference)
≥64	18 (75.0)	25 (47.2)	0.023	3.36 (1.15–9.79)
**Parity**				
<4	16 (66.7)	35 (66.0)		1 (reference)
≥4	8 (33.3)	18 (34.0)	0.96	0.97 (0.35–2.70)
**BMI (kg/m**^**2**^**)**				
Healthy <24	10 (41.7)	31 (58.5)		
Overweight 24–30	14 (58.3)	20 (37.7)		
Obese >30	0	2 (3.8)	0.13	—
**Past history**				
HRT	1 (4.2)	2 (3.8)	1.0	0.90 (0.08–10.46)
Menopause	23 (95.8)	49 (92.5)	1.0	0.53 (0.06–5.04)
H/T	10 (41.7)	22 (41.5)	0.99	0.99 (0.37–2.64)
DM	4 (16.7)	7 (13.2)	0.73	0.76 (0.20–2.89)
Hysterectomy	5 (20.8)	9 (17.0)	0.75	0.77 (0.23–2.63)
Previous POP or SUI surgery	2 (8.3)	5 (9.4)	1.0	1.15 (0.21–6.37)
**Prolapse**				
Stage II	2 (8.3)	8 (15.1)		1 (reference)
Stage III – IV	22 (91.7)	45 (84.9)	0.72	1.96 (0.38–9.99)
Anterior wall prolapse	23 (95.8)	45 (84.9)	0.17	0.24 (0.03–2.08)
Apical prolapse	18 (75.0)	41 (77.4)	0.82	1.14 (0.37–3.51)
Isolated uterine prolapse	0	6 (11.3)	0.17	—
Posterior wall prolapse	5 (20.8)	9 (17.0)	0.75	0.78 (0.23–2.63)
**POP-Q**				
Aa ≥ 2	14 (58.3)	19 (35.9)	0.065	0.40 (0.15–1.07)
Ba ≥ 4	12 (50.0)	15 (28.3)	0.065	0.39 (0.15–1.07)
C ≥ 3	7 (29.2)	15 (28.3)	0.94	0.96 (0.33–2.78)
TvL ≥ 10	11 (45.8)	15 (28.3)	0.13	0.47 (0.17–1.27)

^†^Fisher’s exact test and χ^2^ test were performed for categorical variables.

^‡^The univariate logistic regression was applied for odds ratios and 95% confidence intervals.

^*^OR: odds ratios; CI: confidence intervals; BMI: body mass index; HRT: hormone replacement therapy; H/T: hypertension; DM: diabetes mellitus; POP: pelvic organ prolapse; SUI: stress urinary incontinence.

**Table 5:** Operative methods, operative outcomes and questionnaire in de novo stress urinary incontinence (SUI) group (n = 24) and no SUI group (n = 53).

	De novo SUI group (n = 24)	No SUI group (n = 53)	*p* value^†^	OR (95% CI)^‡^
**Device**				
Anterior mesh	13 (54.2)	31 (58.5)	0.72	1.19 (0.45–3.15)
Anterior and posterior mesh	10 (41.7)	15 (28.3)	0.25	0.55 (0.20–1.51)
Posterior mesh	1 (4.2)	7 (13.2)	0.42	3.50 (0.41–0.17)
Concomitant hysterectomy	13 (54.2)	35 (66.0)	0.32	1.65 (0.62–4.40)
**Pre-operative symptoms**				
Urinary frequency	13 (54.2)	17 (32.1)	0.07	0.40 (0.15–1.07)
UI	9 (37.5)	13 (24.5)	0.24	0.54 (0.19–1.53)
**Urodynamic diagnosis**				
Occult USI	7 (29.2)	2 (3.8)	0.003	10.0 (2.00–50.0)
SUI history	10 (41.7)	4 (7.6)	0.001	9.1 (2.38–33.3)
DO	8 (33.3)	15 (28.3)	0.66	0.79 (0.28–2.23)
BOO	9 (37.5)	23 (43.4)	0.70	1.10 (0.40–3.12)
MUCP drop after reduction	8 (33.3)	16 (30.2)	0.73	0.76 (0.20–2.89)
OABSS ≥ 6	10 (41.7)	17 (32.1)	0.41	0.66 (0.24–1.79)

^†^Fisher’s exact test and χ^2^ test were performed for categorical variables.

^‡^The univariate logistic regression was applied for odds ratios and 95% confidence intervals.

^*^OR: odds ratios; CI: confidence intervals; UI: urgency incontinence; USI: urodynamic stress incontinence; DO: detrusor overactivity; BOO: bladder outlet obstruction; MUCP: maximum urethral closure pressure; OABSS: overactive bladder symptom score.

### The independent predictors of postoperative de novo SUI

Univariate logistic regression was used to determine independent predictors of postoperative de novo SUI. Women older than 64 years of age were 3.36 times (95% CI 1.15–9.79) at a greater risk than younger women; while women who had occult urodynamic stress incontinence and the previous history of SUI were 10.0 times (95% CI 2.0–50.0) and 9.1 times (95% CI 2.38–33.3) at greater risks than no history women, respectively.

## Discussion

This is the first study for evaluating the predictors of de novo SUI following Elevate mesh surgeries. The strongest predictors are age over 64 years old, occult urodynamic stress incontinence and previous history of SUI. The de novo SUI rate after Elevate mesh placement is 31.2%.

Among the studied cohort, over half (87 of 164, 53%) were incontinent women. After exclusion, the prolapse reduction test with vaginal gauze roll during the urodynamic study revealed occult urodynamic stress incontinence in 9 of 77 (12%) of the preoperatively continent women, which is compatible to the rate reported previously^14^. A study compared the detection rates of occult urodynamic stress incontinence with 5 different prolapse reduction methods, and the detection rate ranged from 6% to 30%^15^. But the study did not mention vaginal gauze roll, which can also be applied easily as a prolapse reduction method in daily practice^16^.

It was reported that approximately a quarter of continent women with POP develop de novo SUI after prolapse repair with or without mesh^2,4^. In our study, 31.2% of women developed de novo SUI after Elevate mesh surgeries. Previous studies revealed 26.3% (15 of 57) and 28.1% (18 of 64) de novo SUI after anterior Elevate mesh surgeries, but those studies excluded occult urodynamic stress incontinence before analysis^12,17^. The incidence of de novo SUI after Elevate mesh surgeries is higher than expected according to our result.

Among the 9 women with occult urodynamic stress incontinence, 7 (77.8%) developed de novo SUI. In previous articles, only 65% women with stage III to IV POP and occult urodynamic stress incontinence^18^ and 12.5% women with stage II POP and occult urodynamic stress incontinence^19^ developed de novo SUI after pelvic reconstruction surgeries without concurrent anti-incontinence surgery. The reason that the anterior Elevate mesh surgery resulted in more de novo SUI may be the extensive dissection from paravesical space to sacrospinous ligament bilaterally, which might result in more tissue damage and denervation^17^. Interestingly, previous literature and our study revealed that not all women having positive reduction test for occult SUI develop de novo SUI after pelvic reconstruction surgeries. In our study, 2 remained continent after the Elevate mesh surgeries. The reason may be the reduction test itself that deteriorates the urethral closure mechanism^20^. Therefore, further study is needed to determine the real urethral closure mechanism and evaluate occult urodynamic stress incontinence.

POP surgery can result in an improvement of SUI; meanwhile, it may unmask SUI which had been obscured by POP preoperatively^2^. Kuribayashi and his colleagues had found that preoperative evaluation of urethral obstruction will contribute to the prediction of de novo SUI combined with a conventional diagnosis of occult urodynamic stress incontinence^11^. Besides, a reduction of MUCP was also reported after transvaginal mesh surgery^21^. Theoretically, more women with BOO and reduction of MUCP are supposed to be found in de novo SUI group. However, neither BOO nor reduction of MUCP was found statistically different between the 2 groups in our study, indicating that unmasked BOO and reduction of MUCP cannot explain the mechanism of de novo SUI in the Elevate mesh surgeries. We found that age and previous incontinence history, either self-reported or diagnosed on examinations, are the key predictors for de novo SUI after reconstruction surgery. Firstly, age is an irreversible factor which has a negative impact on urethral closure. Secondly, once incontinence happens, no matter in daily life or in diagnostic tests, it is very likely to occur again after anatomic correction of POP.

On April 16, 2019, the FDA ordered all manufacturers to stop selling synthetic mesh intended for transvaginal repair of anterior compartment prolapse, because the manufacturers have not demonstrated a reasonable assurance of safety and effectiveness for these devices^22^. Although there is going to have a great change in operative methods in POP correction, we still have to look into the advantage and disadvantage of transvaginal mesh from the experience and studies before the announcement of FDA. In POP correction, Elevate mesh provides effective surgical outcome with low mesh extrusion rate^23^ despite a higher incidence of de novo SUI^17^. However, this does not mean that other single-incision meshes will result in a similar surgical outcome. Each commercial kit varies in approach methods, interaction with surrounding tissue, efficacy and adverse effect so that more studies are needed to find out.

Many studies tried to identify predictors of de novo SUI to evaluating the indications of concomitant anti-incontinence surgery. In women with POP and SUI (symptomatic or occult), concurrent MUS may reduce postoperative SUI^6^. Although de novo SUI is not uncommon, the symptoms might not bother some patients too much^17^. It might be feasible to postpone the MUS and perform a two-staged procedure if required^6^. Therefore, the value of de novo SUI prediction is for preoperative counseling of the indications of concomitant anti-incontinence surgery and postoperative education of the symptoms. Concurrent MUS should only be provided to high-risk women who can best benefit from reduced postoperative SUI. Therefore, whether concurrent MUS is needed should be discussed during the preoperative consultation, especially for women over 64-year-old, with a history of SUI or occult urodynamic stress incontinence, when scheduled to receive the Elevate mesh surgeries.

There is no doubt that the retrospective design of this study is a major limitation. Although the analysis was retrospective, all data were collected prospectively in a standard survey for patient care visits. The strength of our study is the simplicity and effectiveness of surgical procedures that all participants underwent the same mesh surgeries. We believe this can minimize the limitation of the study design.

## Patients and Methods

Between November 2011 and February 2014, medical records of all women who received Elevate mesh procedures for their stage II to IV POP without concomitant urinary incontinence at a tertiary referral center at Taiwan were reviewed.

### Approval

This study received approval from the Institutional Review Board of Kaohsiung Medical University Hospital. All basic and experimental data collection was performed in accordance with the International Urogynecological Association (IUGA)/International Continence Society (ICS) joint report on the terminology for female pelvic floor dysfunction^24^. All procedures were performed in accordance with original advice of the Elevate Device (American Medical Systems, Minnetonka, MN).

A total of 164 women were included. Fifty-five women were excluded due to concomitant MUS for preoperatively diagnosed overt or occult SUI. Eighteen women were excluded due to preoperative incontinence without MUS. Another 14 were excluded due to incomplete medical records. Thus, our study examined the de novo SUI rate based on 77 available subject files.

The review of the chart records consisted of a detailed history before and 6 months after the surgery, including urinary analysis, pelvic examination using POP-Q system^24^, urodynamic studies (UDS) and 1-hour pad test, and personal interview to identify urinary symptoms with the Overactive Bladder Symptom Score (OABSS)^25^. Urinary symptoms with the standardized questionnaire taking into account the 2002 ICS definitions^26^. Women were asked to fill out the VAS (visual analog scale) scores during the postoperative day 1 round. Urodynamic studies were performed according to the recommendations by the International Continence Society^27^ with a 6-channel urodynamic monitor (MMS; UD2000, Enschede, Netherlands). The diagnosis of occult urodynamic stress incontinence was made by the occurrence of urinary leakage in the stress test after prolapse reduction with vaginal gauze roll with the strong-desire amount of urine in the bladder. Any uninhibited detrusor contraction during filling cystometry was deemed positive for detrusor overactivity (DO). Bladder outlet obstruction (BOO) was diagnosed by pressure-flow study if the maximum flow (Q_max_) was equal or less than 12 mL/s, and peak detrusor pressure at peak flow (P_detQmax_) was equal or more than 20 cmH_2_O^10,28^.

### Operative technique: Elevate device – anterior/apical mesh

The selection of operative methods depended on the preoperative diagnosis. All surgical procedures were performed under general anesthesia. Every patient received a single dose of intravenous prophylactic antibiotic. The patients were placed in a lithotomy position. After hydro-dissection and separation of the paravesical fascia and vaginal mucosa, the Elevate anterior mesh with bilateral upper and lower arms were pushed to the obturator fascia and sacrospinous ligaments, respectively. Graft anchorage was performed via self-fixating tips. Then, we performed a cystoscopy to check if there is any bladder or ureter injury. The synthetic mesh was put under the bladder base and anchored with 2–0 polydioxanone (PDS) sutures proximally and distally. The vaginal wound was closed with 2–0 Vicryl sutures. The skin wounds were closed using Dermabond, and vaginal packing was placed for 48 hours.

### Operative technique: Elevate device – posterior mesh

After hydro-dissection, a posterior longitudinal incision was created and bilateral sacrospinous ligaments were identified. The posterior mesh implant arms with hooks were anchored in the sacrospinous ligament. Then another 2-arm synthetic mesh was pushed inside as deep as possible. Graft anchorage was performed via self-fixating tips. PDS 2–0 was sutured over cervical stroma or vaginal vault with mesh. The vaginal wound was closed with 2–0 Vicryl sutures, and vaginal packing was placed for 48 hours.

### Informed consent

Informed consent was obtained from all participants before the surgeries.

### Statistics

IBM SPSS Statistical Software version 20.0 ed. was used for statistical analyses. The Wilcoxon signed-rank test was performed for comparison between pre-operative and post-operative POP-Q parameters. Fisher’s exact test and χ^2^ test were performed for categorical variables. The Univariate logistic regression was applied for odds ratios and 95% confidence intervals. A *p-*value of less than 0.05 was considered statistically significant.

## Conclusions

Age over 64 years old, occult urodynamic stress incontinence and previous history of SUI are 3 significant predictors for de novo SUI following the single-incision mesh surgeries. For high-risk women, whether a concurrent MUS is needed should be discussed during the preoperative consultation.

## Data Availability

The datasets generated during and analyzed during the current study are available from the corresponding author on reasonable request.
